# Dynamic early warning scores for predicting clinical deterioration in patients with respiratory disease

**DOI:** 10.1186/s12931-022-02130-6

**Published:** 2022-08-11

**Authors:** Sherif Gonem, Adam Taylor, Grazziela Figueredo, Sarah Forster, Philip Quinlan, Jonathan M. Garibaldi, Tricia M. McKeever, Dominick Shaw

**Affiliations:** 1 Department of Respiratory Medicine, Nottingham City Hospital, Nottingham University Hospitals NHS Trust, Hucknall Road, Nottingham, NG5 1PB UK; 2 NIHR Nottingham Biomedical Research Centre, School of Medicine, University of Nottingham, Nottingham, UK; 3 Digital Research Service, University of Nottingham, Nottingham, UK; 4 School of Computer Science, University of Nottingham, Nottingham, UK

**Keywords:** Early warning score, Risk prediction, Clinical deterioration

## Abstract

**Background:**

The National Early Warning Score-2 (NEWS-2) is used to detect patient deterioration in UK hospitals but fails to take account of the detailed granularity or temporal trends in clinical observations. We used data-driven methods to develop dynamic early warning scores (DEWS) to address these deficiencies, and tested their accuracy in patients with respiratory disease for predicting (1) death or intensive care unit admission, occurring within 24 h (D/ICU), and (2) clinically significant deterioration requiring urgent intervention, occurring within 4 h (CSD).

**Methods:**

Clinical observations data were extracted from electronic records for 31,590 respiratory in-patient episodes from April 2015 to December 2020 at a large acute NHS Trust. The timing of D/ICU was extracted for all episodes. 1100 in-patient episodes were annotated manually to record the timing of CSD, defined as a specific event requiring a change in treatment. Time series features were entered into logistic regression models to derive DEWS for each of the clinical outcomes. Area under the receiver operating characteristic curve (AUROC) was the primary measure of model accuracy.

**Results:**

AUROC (95% confidence interval) for predicting D/ICU was 0.857 (0.852–0.862) for NEWS-2 and 0.906 (0.899–0.914) for DEWS in the validation data. AUROC for predicting CSD was 0.829 (0.817–0.842) for NEWS-2 and 0.877 (0.862–0.892) for DEWS. NEWS-2 ≥ 5 had sensitivity of 88.2% and specificity of 54.2% for predicting CSD, while DEWS ≥ 0.021 had higher sensitivity of 93.6% and approximately the same specificity of 54.3% for the same outcome. Using these cut-offs, 315 out of 347 (90.8%) CSD events were detected by both NEWS-2 and DEWS, at the time of the event or within the previous 4 h; 12 (3.5%) were detected by DEWS but not by NEWS-2, while 4 (1.2%) were detected by NEWS-2 but not by DEWS; 16 (4.6%) were not detected by either scoring system.

**Conclusion:**

We have developed DEWS that display greater accuracy than NEWS-2 for predicting clinical deterioration events in patients with respiratory disease. Prospective validation studies are required to assess whether DEWS can be used to reduce missed deteriorations and false alarms in real-life clinical settings.

**Supplementary Information:**

The online version contains supplementary material available at 10.1186/s12931-022-02130-6.

## Introduction

Early warning scores are used to detect deteriorating patients in acute hospital settings, and are usually calculated by assigning scores to a number of clinical observations such as heart rate and respiratory rate, and adding these to produce a composite score [1–3]. The National Early Warning Score-2 (NEWS-2) is used throughout the UK and internationally [4]. NEWS-2 is simple enough to be used with paper observation charts and calculated by hand, but this may result in useful diagnostic information being lost, as the detailed granularity and temporal trends in clinical observations are not accounted for. For instance, NEWS-2 has only two categories for inspired oxygen, whereas it has been shown that incorporating the percentage of inspired oxygen into scoring systems improves their accuracy [5]. The increasing use of electronic recording of clinical observations raises the possibility of using more sophisticated scoring systems that make full use of the information content of current and previous observations. There is increasing interest in using advanced statistical methods to train and validate novel scoring systems, making use of large datasets of clinical observations [6].

The majority of early warning scoring systems have been developed and validated to predict intensive care unit (ICU) admission, cardiac arrest or death [1–3]. However, the purpose of an early warning score is to detect patients who require urgent intervention in order to *prevent* these adverse outcomes, rather than simply to predict them. There are few previous studies that have developed and validated an early warning score specifically to detect treatable conditions such as sepsis and respiratory failure. We therefore defined a novel outcome of clinically significant deterioration (CSD) requiring urgent treatment, and utilised this to develop and validate a novel early warning score.

We developed and internally validated dynamic early warning scores (DEWS) using a retrospective database of clinical observations in patients admitted under the care of adult respiratory medicine services. We hypothesised that DEWS would provide superior predictive accuracy compared to NEWS-2 in patients with respiratory disease, with respect to (1) death or ICU admission, occurring within 24 h (D/ICU), and (2) clinically significant deterioration requiring urgent treatment, occurring within 4 h (CSD).

### Data source

The study population comprised adult patients (age ≥ 18 years) admitted between 1st April 2015 and 31st December 2020 who were under the care of respiratory medicine at the time of death or discharge from hospital. The majority of patients had an acute or chronic respiratory diagnosis although some general medical patients were also included if they were cared for on a respiratory ward.

Clinical observations for adult in-patients at Nottingham University Hospitals NHS Trust (NUH) have been recorded electronically using a wireless workflow tracking system since April 2015 as part of routine clinical care. Clinical observations data were extracted from the system for the study population. The data comprised date and time-stamped measurements of heart rate, respiratory rate, systolic blood pressure, temperature, oxygen saturations, inspired oxygen flow rate or concentration (FiO_2_), and level of consciousness recorded on a five-point ACVPU scale (Alert, Confused, responds to Voice, responds to Pain, Unresponsive). The NEWS-2 score was calculated according to current guidelines [4]. Patients in whom at least one observation set was labelled as “O2 sats scale 2 (chronic respiratory disease)” were considered to have chronic respiratory disease, with target oxygen saturations of 88–92%. Oxygen saturation Scale 2 was used to calculate NEWS-2 in these patients; Scale 1 was used for all other patients. The timing of death or ICU admission was also extracted from the system.

### Clinically significant deterioration (CSD) definition

A subset of 1100 admission episodes were annotated manually by a Consultant Physician and senior Specialty Registrar (SG and SF) with reference to the medical notes. Clinically significant deterioration was defined as a specific event requiring a change in treatment. In order to ensure consistency within and between the case annotators, the types of event and treatments given were recorded using a standardised list, as shown in Additional file 1: Table S1. The list of event types and treatments was drafted based on clinical experience and previously published literature [7–11], and was finalised following preliminary annotation of 50 cases by the lead investigator (SG). Ten cases were reviewed jointly by SG and SF in order to agree a consistent approach to annotation. To maximise the number of events available for analysis, the cases chosen for annotation were those with a maximum NEWS-2 score of ≥ 10, or in which death or ICU admission occurred. In addition, since events with a low heart rate were uncommon in the dataset, all admission episodes with a minimum heart rate of ≤ 40 were annotated, to ensure sufficient training examples for this rare but important condition.

The dataset was anonymised prior to analysis by removing identifying information such as names, dates of birth and hospital identification numbers. The project was approved by the Nottingham 1 Research Ethics Committee (20/EM/0064) and the Confidentiality Advisory Group (20/CAG/0034).

### Model development and validation

For the full dataset with the outcome of D/ICU, data from April 2015 to December 2019 were used for model training. Data from January to December 2020 were then extracted and used for validation. For the annotated dataset with the outcome of CSD, 829 randomly selected admission episodes were used for training and 271 for validation. Since the missing data rate was low (< 1% for each variable), the analysis was limited to complete cases and data imputation was not carried out. The first two observation sets from each admission episode were excluded from the analysis since (1) our primary aim was to detect de novo deterioration occurring during the admission rather than to stratify illness severity at the point of admission, and (2) a number of time series features included in the DEWS model required a minimum of three observation sets to calculate.

DEWS was developed using similar methodology to the previously published logistic early warning score (logEWS) [12] and Dynamic individual vital sign trajectory early warning score (DyniEWS) [13]. Since the level of inspired oxygen had mixed units of measurement (percentage inspired oxygen and flow rate in litres/minute) we created a new ordinal variable which encoded the level of inspired oxygen as None = 0, Low = 1, Low-moderate = 2, Moderate = 3, High = 4, and Very high = 5. Full details of this encoding are shown in Additional file 1: Table S2. Clinical observations with a U-shaped risk curve, in which both high and low values were associated with increased risk (heart rate, respiratory rate, systolic blood pressure and temperature) were split into separate variables for high and low values (see Additional file 1: Table S3). A number of time series features were extracted from the raw clinical observations data including: difference from the previous observation; average and standard deviation of the five (minimum of three) most recent observations; and categorisation of recent values into normal and stable, normal and unstable, outside normal range and stable, outside normal range and improving, or outside normal range and worsening. A total of 38 raw and engineered features were entered into logistic regression models, with L2 regularisation for feature selection, and tenfold stratified cross-validation. The output of the logistic regression models was the modelled probability of the outcome. All features were normalised to zero mean and unit variance prior to entry into the models. Separate DEWS were developed for the outcome of D/ICU in the full dataset and CSD in the annotated dataset. Further details of the DEWS models are given in the supplementary material (Additional file 1: Tables S2–S6).

The primary metric of model accuracy was the area under the receiver operating characteristic curve (AUROC). The area under the precision-recall curve (AUPRC) was also calculated since this is considered to be a more informative metric in unbalanced datasets with a large majority of negative cases [14]. Precision-recall curves are helpful in these cases as they give an intuitive understanding of how the precision (also known as the positive predictive value, the probability that a positive test result is a true positive) relates to the recall (or sensitivity) at different cut-points. Area under the curve values and 95% confidence intervals were calculated using 500 bootstrap samples. The sensitivity and specificity of NEWS-2 and DEWS were compared at cut-points corresponding to NEWS-2 scores of 5 and 7, since these are the key thresholds for an urgent or emergency response in current guidelines [4].

### Sample size calculation

We used a previously published method [15] to calculate the required sample size for comparing the AUROC of two diagnostic tests, in order to determine how many cases needed to be manually annotated. The AUROC of NEWS for predicting in-hospital death or unplanned ICU admission is approximately 0.8 [5]. Assuming that the novel algorithm would improve this to 0.85, we calculated that 463 observation sets positive for the outcome would be needed in the validation dataset to detect this difference with 80% power. We estimated that this would be achieved if 250 admission episodes were included in the validation dataset. It is usually recommended that the training dataset is 2–4 times the size of the validation dataset, so we planned to annotate a further 750 admission episodes for the training dataset.

## Results

Data were extracted for 31,590 admission episodes, mean (standard deviation) age 66.2 (17.2) years, 52.9% female, consisting of 1,037,349 rows of date and time-stamped clinical observation sets, of which 1,025,611 (98.9%) were complete. Following removal of incomplete observation sets, and exclusion of the first two observation sets of each admission episode, 963,561 observation sets remained for model training and validation. Dataset characteristics are given in Table 1 and summary statistics for each of the raw clinical observations are shown in Additional file 1: Table S7.

**Table 1:** Dataset characteristics

	Full dataset (training)	Full dataset (validation)	Annotated dataset (training)	Annotated dataset (validation)
Admission episodes	26,470	5120	829	271
Age (mean [SD])	66.3 (17.2)	65.6 (16.8)	66.6 (15.0)	65.6 (16.4)
Female (n [%])	14,172 (53.5)	2540 (49.6)	440 (53.1)	135 (49.8)
Mortality (n [%])	1616 (6.1)	425 (8.3)	173 (20.9)	27 (10.0)
ICU admission (n [%])	648 (2.4)	311 (6.1)	362 (43.7)	71 (26.2)
Observation sets	787,662	175,899	52,803	16,830
NEWS-2 score (mean [SD])	3.5 (2.5)	3.0 (2.1)	4.6 (2.7)	4.6 (2.7)
Annotated CSD events	–	–	1036	347
Observations sets positive for outcome (n [%])*	16,726 (2.1)	5358 (3.0)	2840 (5.4)	951 (5.7)

*SD* standard deviation, *ICU* intensive care unit, *CSD* clinically significant deterioration

*Outcome was death or intensive care unit admission occurring within 24 h for full dataset; and clinically significant deterioration occurring within 4 h for annotated dataset

**Fig. 1:**
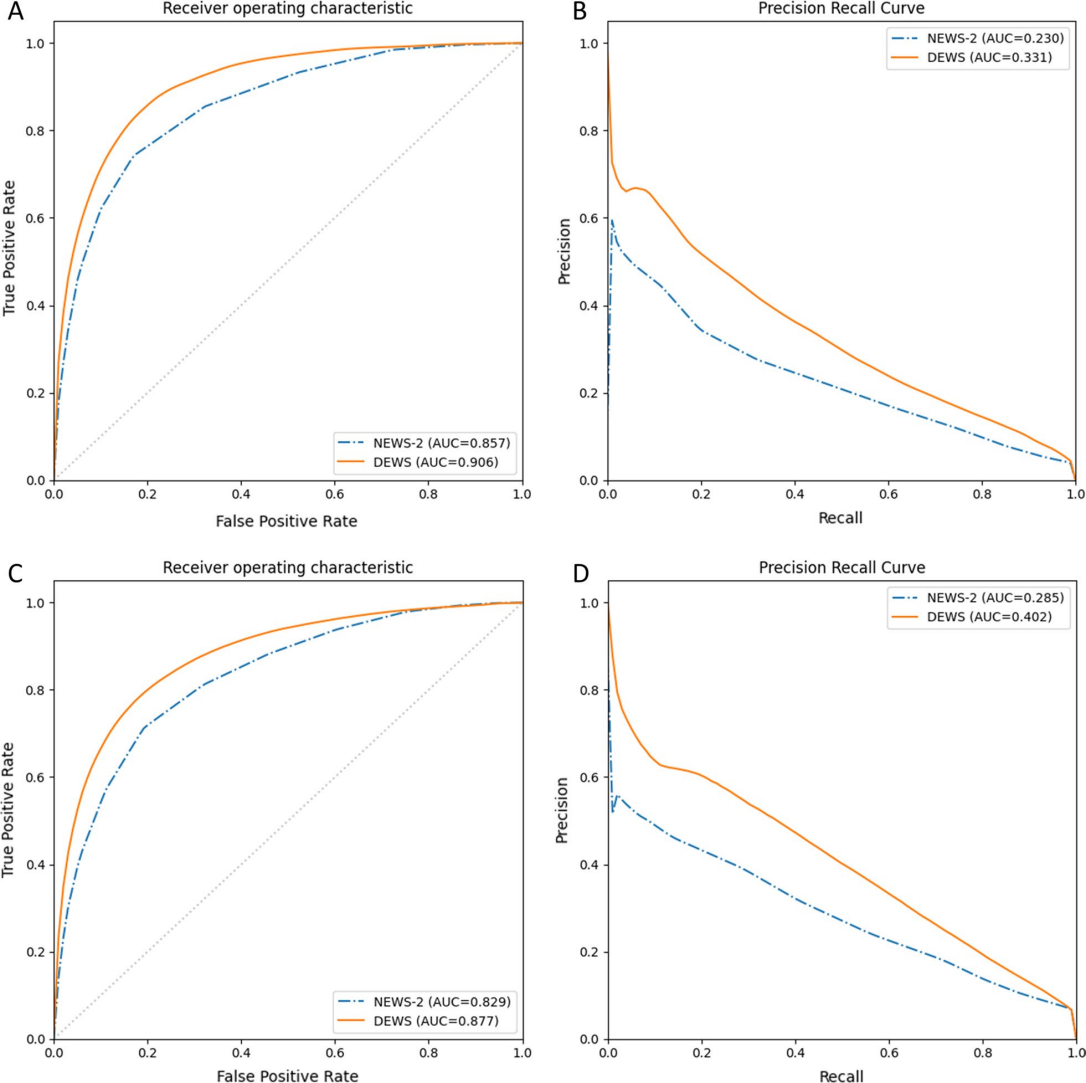
Receiver operating characteristic and precision-recall curves for the prediction of clinical outcomes in the validation datasets. **A** and **B** show receiver operating characteristic and precision-recall curves respectively, for the prediction of death or intensive care unit admission occurring within 24 h. **C** and **D** show the equivalent curves for the prediction of clinically significant deterioration occurring within 4 h

DEWS demonstrated better areas under the curve than NEWS-2 for both outcomes (Fig. 1 and Table 2). Tables 3 and 4 show the sensitivity and specificity of NEWS-2 and DEWS, at NEWS-2 cut-offs of ≥ 5 and ≥ 7 and DEWS cut-offs with matched sensitivity and specificity, in the validation datasets for the outcomes of D/ICU and CSD respectively. DEWS was associated with reductions in false positive (false alarm) and false negative (missed deterioration) rates in comparison to NEWS-2 for both outcomes. The annotated validation dataset contained a total of 347 CSD events. Using a NEWS-2 cut-off of ≥ 5 and a DEWS cut-off with equivalent specificity (≥ 0.021), 315 (90.8%) of these were detected by both NEWS-2 and DEWS, at the time of the event or within the previous 4 h; 12 (3.5%) were detected by DEWS but not by NEWS-2, while 4 (1.2%) were detected by NEWS-2 but not by DEWS; 16 (4.6%) were not detected by either scoring system.

**Table 2:** Area under the receiver operating characteristic and precision-recall curves for the prediction of clinical outcomes by NEWS-2 and DEWS

Outcome (Dataset)	NEWS-2 AUROC (95% CI)	DEWS AUROC (95% CI)	NEWS-2 AUPRC (95% CI)	DEWS AUPRC (95% CI)
D/ICU (training data)	0.865 (0.862–0.868)	0.902 (0.893–0.910)	0.206 (0.199–0.212)	0.290 (0.263–0.317)
D/ICU (validation data)	0.857 (0.852–0.862)	0.906 (0.899–0.914)	0.230 (0.219–0.242)	0.331 (0.301–0.359)
CSD (training data)	0.817 (0.809–0.825)	0.857 (0.837–0.872)	0.263 (0.247–0.279)	0.323 (0.266–0.377)
CSD (validation data)	0.829 (0.817–0.842)	0.877 (0.862–0.892)	0.285 (0.259–0.318)	0.402 (0.346–0.455)

*NEWS-2* National Early Warning Score-2, *DEWS* dynamic early warning score, *AUROC* area under the receiver operating characteristic curve, *AUPRC* area under the precision-recall curve, *CI* confidence interval, *D/ICU* death or intensive care unit admission, occurring within 24 h, *CSD* clinically significant deterioration, occurring within 4 h

**Table 3:** Sensitivity and specificity of NEWS-2 and DEWS for predicting death or ICU admission within 24 h in the validation dataset

	Sensitivity (%)	Specificity (%)	False positive rate*	False negative rate*
NEWS-2 ≥ 5	74.2	83.0	16.5	0.79
DEWS ≥ 0.030	74.5	88.5	11.1	0.78
DEWS ≥ 0.020	82.3	83.3	16.2	0.54
NEWS-2 ≥ 7	47.3	94.7	5.2	1.61
DEWS ≥ 0.094	47.2	96.8	3.1	1.61
DEWS ≥ 0.062	57.5	94.7	5.2	1.30

*Percentage of all observation sets

**Table 4:** Sensitivity and specificity of NEWS-2 and DEWS for predicting clinically significant deterioration within 4 h in the validation dataset

	Sensitivity (%)	Specificity (%)	False positive rate*	False negative rate*
NEWS-2 ≥ 5	88.2	54.2	43.2	0.67
DEWS ≥ 0.032	88.3	69.2	29.0	0.66
DEWS ≥ 0.021	93.6	54.3	43.1	0.36
NEWS-2 ≥ 7	71.4	80.8	18.1	1.62
DEWS ≥ 0.073	71.5	88.0	11.4	1.61
DEWS ≥ 0.050	80.0	80.8	18.2	1.13

*Percentage of all observation sets

Additional file 1: Tables S8 and S9 show the multivariate logistic regression coefficients for each of the 38 features included in the DEWS models for predicting D/ICU and CSD respectively. High heart rate and respiratory rate, and low systolic blood pressure were strong features of increased risk in both cases, as were the slope category features for heart rate and respiratory rate. Inspired oxygen was a stronger predictor of risk than oxygen saturations, for both outcomes. Low temperature was a strong predictor of D/ICU but less so for CSD. In the CSD model, the feature coefficient for heart rate rolling average was negative, whereas that for high heart rate it was positive. This caused the two features to act in opposite directions, but with the overall effect that high heart rates had less effect on DEWS if the baseline heart rate was also high. A similar effect was seen with systolic blood pressure in the CSD model, with low blood pressure having less effect on DEWS if the baseline blood pressure was also low.

## Discussion

### Main findings

We have described a novel outcome measure (clinically significant deterioration [CSD]) for the development of early warning scores, which may be considered of greater clinical relevance than death or ICU admission, since it captures events which are potentially treatable and reversible. We used this outcome, as well as the more traditional composite outcome of death and ICU admission, to train and validate novel dynamic early warning scores which take account of time series features such as trends over time and variability. We showed that DEWS has superior predictive accuracy compared to NEWS-2 for both outcomes, and that DEWS can result in clinically important reductions in false alarms or missed deteriorations compared to NEWS-2. The logistic regression model underlying DEWS lends itself to interpretability, since it does not rely on opaque neural networks or other complex models. A qualitative comparison of the regression coefficients shows that there are similarities but also important differences between the models trained on the two outcomes.

### Results in the context of previous research

A number of previous studies have used machine learning or advanced statistical methods to predict patient deterioration [6]. These vary widely with respect to the patient cohorts studied, data analysis methods used, predictor variables entered into the model and outcomes to be predicted. Our methods were closest to those of Zhu et al. [13] who aimed to predict death, cardiac arrest or unplanned ICU admission in patients post-cardiac surgery using a Dynamic individual vital sign trajectory early warning score (DyniEWS). These investigators reported an AUROC of 0.80 for DyniEWS compared to 0.73 for NEWS, similar to the improvements we saw for DEWS compared to NEWS-2. More recently, Pimental et al. developed and implemented a model (Hospital-wide Alerting Via Electronic Noticeboard [HAVEN]) for predicting cardiac arrest or unplanned ICU admission, using more complex machine learning methods (gradient boosted trees) and a large number of predictors including laboratory blood tests [16]. HAVEN achieved an AUROC of 0.901 for the primary outcome compared to 0.842 for NEWS. However, a disadvantage of the HAVEN model is its complexity, which could reduce model interpretability for clinicians and make widespread adoption more challenging. We are aware of one previous study that trained a predictive model using manually annotated events. Blackwell et al. [17] reviewed the medical notes of 457 patients who were transferred to ICU from an acute cardiac unit due to a clinical deterioration, and classified the reason for transfer into one of seven categories. They developed separate logistic regression models for predicting each of these causes of deterioration, as well as a combined model for predicting any event.

### Strengths and limitations

This is one of the first studies to have developed and validated an early warning score to predict clinician-defined deterioration requiring treatment—despite the fact that detecting these events is the underlying purpose of early warning scores. Other than the study of Blackwell et al. [17], early warning scores have usually been developed and validated using outcomes such as ICU admission, cardiac arrest or death [1–3], since these could be automatically extracted from administrative databases without the need for manual annotation by clinical experts. This relies on the unproven and possibly incorrect assumption that the physiological changes preceding these surrogate events are identical to those preceding treatable conditions such as sepsis or respiratory failure. This issue has been recognised by a number of previous authors, acknowledging the difficulty of reliably capturing deteriorations that lead to ward-based interventions [1, 2, 13, 16].

This was a retrospective study which employed previously collected healthcare data, and was limited to patients being cared for on general respiratory wards within a single centre. Therefore it is not yet known whether the DEWS models we have developed have more general applicability. However, many of the deterioration events that occurred in our patient cohort (such as sepsis and cardiac arrhythmias) are similar to what may be expected to occur in other patient groups, such as general medical in-patients. Further studies are needed to generalise our results in other patient groups and healthcare settings. A further limitation was that in order to ensure adequate numbers of CSD events for analysis, we selected cases for annotation with a maximum NEWS-2 score of ≥ 10, or in which death or ICU admission occurred, so that patients included in the annotated dataset had a higher event probability than the study population as a whole. This means that the positive and negative predictive values derived from the annotated dataset are not generalisable to our whole study population, but the sensitivity and specificity values remain valid since these are not affected by the underlying event probability.

### Future work

Although the DEWS we developed are statistically superior to NEWS-2, it is not yet known whether this will translate to a real-world reduction in unnecessary medical reviews or failure-to-rescue events. This is because out-of-hours alerts generated by high NEWS-2 scores at our institution are already subject to review and possible de-escalation by ward nursing staff and hospital coordinators, and on the other hand nurses can request medical review even when the NEWS-2 alert threshold has not been reached. Indeed, previous studies have shown that filtering of alerts, for instance by a team of specialist nurses, appears to be essential to avoid overloading the rapid response team [18–22]. Observational and qualitative studies are needed to determine how NEWS-2 and other early warning scores inform decision-making in real-life settings. This will help to determine whether and how best to implement novel scores such as DEWS. Furthermore, since DEWS has so far only been validated in patients on respiratory wards in a single centre, we plan to undertake external validation in other hospitals and in broader patient populations.

An important aspect of this study was the inclusion of clinician-defined deterioration requiring treatment as an outcome to be predicted, but manually extracting this information from the medical notes is a labour-intensive process. Future studies should investigate alternative methods of capturing ward-based deteriorations, for instance by using natural language processing of electronic health records, electronic prescribing data, or real-time feedback from frontline clinicians.

Interpretability of machine learning models is critical to gaining the trust of clinicians and is a growing field of research [23]. Lauritsen et al. recently developed explainable machine learning models for predicting sepsis, acute kidney injury, and acute lung injury in unselected acute admissions [24]. The DEWS we have developed use logistic regression, which is a highly transparent modelling framework, and it is straightforward to determine the relative contribution of the features to the model output for any given set of clinical observations. We plan to further refine the interpretability of our model by developing natural language explanations which will allow clinicians to more effectively prioritise patients for urgent medical review.

## Conclusions

This study developed and validated dynamic early warning scores which display superior accuracy compared to NEWS-2 for detecting clinical deterioration in respiratory patients. Prospective observational and interventional studies are needed to evaluate the real-life effectiveness of DEWS and to overcome the technical and organisational challenges of implementing it within complex healthcare systems.

## Supplementary Information

**Additional file 1.** Supplementary methods and results.

## Data Availability

Individual patient-level data used in this study are owned by Nottingham University Hospitals NHS Trust (NUH) and cannot be made generally available due to restrictions on permitted data analysis and data sharing within the approved study protocol and data protection impact assessment. Requests for access to anonymised data will be considered by the authors upon submission of a research proposal to the corresponding author (sherif.gonem@nottingham.ac.uk). If approved, this will be subject to a data sharing agreement between NUH and the proposing institution, as well as submission of study protocol amendments to the Health Research Authority and Confidentiality Advisory Group.

## Abbreviations

ACVPU: Alert, confused, responds to voice, responds to pain, unresponsive
AUPRC: Area under the precision-recall curve
AUROC: Area under the receiver operating characteristic curve
CSD: Clinically significant deterioration
D/ICU: Death or intensive care admission
DEWS: Dynamic early warning score
DyniEWS: Dynamic individual vital sign trajectory early warning score
HAVEN: Hospital-wide alerting via electronic noticeboard
ICU: Intensive care unit
LogEWS: Logistic Early Warning Score
NEWS-2: National Early Warning Score-2
NUH: Nottingham University Hospitals NHS Trust
