# Supplementary material for: Dynamic early warning scores for predicting clinical deterioration in patients with respiratory disease
Source: Respir Res. 2022 Aug 11;23:203. doi: 10.1186/s12931-022-02130-6 (PMC9367123; doi:10.1186/s12931-022-02130-6)
Supplement: Supplementary file 1 — Additional file 1. Supplementary methods and results. [file 12931_2022_2130_MOESM1_ESM.docx]

**Dynamic Early Warning Score for predicting clinical deterioration in patients with respiratory disease**

^1,2^Sherif Gonem

^3^Adam Taylor

^3,4^Grazziela Figueredo

^2^Sarah Forster

^3^Philip Quinlan

^4^Jonathan M Garibaldi

^2^Tricia M McKeever

^1,2^Dominick Shaw

^1^Department of Respiratory Medicine, Nottingham University Hospitals NHS Trust, Nottingham, UK

^2^NIHR Nottingham Biomedical Research Centre, School of Medicine, University of Nottingham, Nottingham, UK

^3^Digital Research Service, University of Nottingham, Nottingham, UK

^4^School of Computer Science, University of Nottingham, Nottingham, UK

Address for correspondence:

Dr. Sherif Gonem

Department of Respiratory Medicine

Nottingham City Hospital

Hucknall Road

Nottingham

NG5 1PB, UK

Tel: +44 115 969 1169

E-mail: [sherif.gonem@nottingham.ac.uk](mailto:sherif.gonem@nottingham.ac.uk)

**Supplementary methods**

*Clinical dataset*

Clinical observations were recorded as part of routine clinical care at Nottingham University Hospitals NHS Trust (NUH). Each record was time-stamped and consisted of 7 key clinical observations: temperature, systolic blood pressure, heart rate, respiratory rate, oxygen saturations, level of consciousness recorded on a five-point ACVPU scale (Alert, Confused, responds to Voice, responds to Pain, Unresponsive), and inspired oxygen (measured either as a flow rate in litres or as a percentage of inspired oxygen). Incidence of death or Intensive Care Unit (ICU) admission was also extracted as an outcome variable alongside a time-stamp. Episodes of clinically significant deterioration requiring treatment were manually extracted from medical case-notes by two of the clinician investigators (SG and SF). Table S1 shows the event types and treatments that were captured.

**Table S1: Types of event and treatments recorded during case annotation**

| **Types of event** | **Treatments** | |
| --- | --- | --- |
| Cardiac arrest  Respiratory  Cardiovascular  Neurological  Sepsis  Other | Cardio-pulmonary resuscitation  Invasive ventilation  Non-invasive ventilation  ICU admission  IV fluids  Antibiotics  Corticosteroids  Bronchodilators  Diuretics | Anticoagulation  Antiarrhythmic  Vasoactive agent  Anticonvulsant or sedative  Interventional procedure  Physiotherapy  Palliation  Other |

*Data analysis packages and code availability*

The complete pipeline of data pre-processing, feature engineering, predictive modelling and evaluation is built with python (3.8) and available in full at: [https://github.com/atayls/icu_data_analysis](https://github.com/ATayls/ICU_data_analysis)

Within the pipeline we utilised three main python packages, pandas (1.2) for data engineering, tsfresh (0.18.0) for timeseries feature extraction and scikit-learn (0.23) for modelling and evaluation.

*Data pre-processing*

(i) Patient observation sets that contained any missing values were removed from the training and validation datasets prior to analysis. Due to an extremely low prevalence of missing values we opted to remove incomplete observation sets to negate the need for any variable imputation; 98.9% of observation sets were kept across all datasets after removal of incomplete sets.

(ii) As the raw variable “Inspired O2” contained mixed units of measurement (percentage inspired oxygen and flow rate in litres/minute) we created a new ordinal variable named “Inspired_O2_Cat” which encoded the level of inspired oxygen as None = 0, Low = 1, Low-moderate = 2, Moderate = 3, High = 4, and Very high = 5. Full details of this encoding are shown in Table S2.

**Table S2: Encoding of inspired oxygen categories**

| **Inspired oxygen category** | **Numerical encoding** | **Inspired oxygen (%) range** | **Inspired oxygen (L/min) range** |
| --- | --- | --- | --- |
| None | 0 | 21 | 0 |
| Low | 1 | 22 to 24 | 0.5 to 2.5 |
| Low-moderate | 2 | 25 to 28 | 3 to 4 |
| Moderate | 3 | 29 to 35 | 5 to 9 |
| High | 4 | 36 to 50 | 10 to 14 |
| Very high | 5 | 51 to 100 | ≥ 15 |

(iii) The raw ACVPU measurement is an ordinal variable to describe patient consciousness; it was encoded to represent this information in numerical form, as Alert = 0; Confused = 1; responds to Voice = 1; responds to Pain = 2; Unresponsive = 3. C and V were given the same score as they are often used interchangeably in clinical practice, and we found that they carried approximately the same risk of patient deterioration in univariate analyses.

(iv) Four out of the initial 7 raw clinical observations had non-monotonic (U-shaped) risk profiles, namely temperature, systolic blood pressure, heart rate and respiratory rate. To avoid breaching the linearity assumption of logistic regression, two new features were created for each of these clinical observations, reflecting the positive distance from the upper end and the negative distance from the lower end of the National Early Warning Score-2 (NEWS-2) zero-scoring range, as shown in Table S3.

**Table S3: Splitting clinical observations with non-monotonic effects**

| **Split clinical observation** | **NEWS2 zero-scoring range** | **Calculation** |
| --- | --- | --- |
| TEMPERATURE_POS | 36.1 to 38 | *max (0, x – 38)* |
| TEMPERATURE_NEG |  | *abs (min (0, x – 36.1))* |
| SYSTOLIC_BP_POS | 111 to 219 | *max (0, x – 219)* |
| SYSTOLIC_BP_NEG |  | *abs (min (0, x – 111))* |
| HEART_RATE_POS | 51 to 90 | *max (0, x – 90)* |
| HEART_RATE_NEG |  | *abs (min (0, x – 51))* |
| RESP_RATE_POS | 12 to 20 | *max (0, x – 20)* |
| RESP_RATE_NEG |  | *abs (min (0, x – 12))* |

*Feature engineering*

To capture the longitudinal dynamics of the clinical observations we created an additional 4 time-based features (see Table S4) for each of temperature, systolic blood pressure, heart rate, respiratory rate, oxygen saturations, inspired O2 category, and ACVPU (numerically encoded as above), with the exception that the SLOPECAT feature was not calculated for ACVPU. The number of prior observations to calculate time series features was treated as a hyper-parameter to optimise, and selected as five based on training set cross-validated performance. Calculations included the five most recent measurements and in some cases required a minimum of 3 valid observations if less than 5 were available (see Table S4). In order to avoid introducing bias, the first two observation sets of each admission episode were not used for model training or validation, but they were used to calculate time series features for the third observation set onwards.

**Table S4: Engineered longitudinal features calculated from raw clinical observations**

| **Engineered Feature** | **Variable Suffix** | **Description** |
| --- | --- | --- |
| Difference | DIFF | Difference from previous observation |
| Rolling average | ROLAVG | Average of last 5 observations (no minimum) |
| Rolling standard deviation | ROLSTD | Standard deviation of last 5 observations (minimum of 3) |
| Slope category | SLOPECAT | Category of slope as defined in Tables S5 and S6 and text below |

Slope category (SLOPECAT) is an ordinal feature which categorises the current value and slope of clinical observations according to the rules described in Table S5. Higher values of SLOPECAT are generally associated with increased risk. Measurements that are outside the NEWS-2 zero-scoring range and have a worsening trajectory map to the highest value, while stable measurements within the NEWS-2 zero-scoring range are assigned the lowest value. For example, a heart rate of 100bpm that has an increasing slope of 20bpm/24hrs would be assigned to category 4 as it is outside the NEWS-2 zero-scoring range and is worsening over the last 24 hours. Alternatively, the same 100bpm measurement with a slope of -5 would result in a category of 2. The bounds used to define stability of the 24 hour slope for each clinical observation are shown in Table S6; these were determined based on clinical experience and examination of the distribution of slopes within the training dataset.

**Table S5: Calculation of slope category feature**

| **NEWS-2 category** | **Slope Stability (see Table S5)** | **Assigned category** |
| --- | --- | --- |
| Within NEWS-2 zero-scoring range | Stable | 0 |
| Within NEWS-2 zero-scoring range | Significant positive or negative trend | 1 |
| Outside NEWS-2 zero-scoring range | Stable | 2 |
| Outside NEWS-2 zero-scoring range | Significant trend towards normal range | 3 |
| Outside NEWS-2 zero-scoring range | Significant trend away from normal range | 4 |

**Table S6: Clinically defined stable slope range for each clinical observation**

| **Clinical observation** | **Lower limit of stable slope range*** | **Upper limit of stable slope range*** |
| --- | --- | --- |
| Heart rate | -10 | 10 |
| Respiratory rate | -2 | 2 |
| Systolic blood pressure | -10 | 10 |
| Temperature | -0.5 | 0.5 |
| Oxygen saturation | -2 | 2 |
| Inspired oxygen category | -1 | 1 |

*Slopes are expressed as change per 24 hours

*Model development and validation*

Prior to model fitting, all features were standardised to zero mean and unit variance. We fitted logistic regression models to predict the outcome variables (i) death or intensive care unit admission, occurring within 24 hours (D/ICU), and (ii) clinically significant deterioration requiring urgent intervention, occurring within 4 hours (CSD), using the 38 raw and engineered features. We used a form of logistic regression known as ridge regression where L2 regularisation penalises predictors that are uninformative and can address the multicollinearity that may be present between the features. This was implemented using the scikit-learn python package v0.23. To better estimate the model’s ability to generalise past the training set, the full training process was encapsulated in a 10-fold grouped stratified cross-validation process, with grouping at the patient level.

To evaluate the performance of the models in the validation datasets we calculated area under the receiver operating characteristic and area under the precision recall curve, with 95% confidence intervals calculated using 500 bootstrap samples.

**Supplement Reference**

1. Zhu Y, Chiu Y-D, Villar SS, Brand JW, Patteril MV, Morrice DJ, Clayton J, Mackay JH. Dynamic individual vital sign trajectory early warning score (DyniEWS) versus snapshot national early warning score (NEWS) for predicting postoperative deterioration. *Resuscitation*. 2020; 157: 176-184.

**Supplementary results**

**Table S7: Summary statistics of clinical observations per analysed dataset**

|  |  | **TEMP** | **SYSTOLIC BP** | **HEART RATE** | **RESP RATE** | **O2 SATS** | **ACVPU ENCODED** | **INSP O2 LITRES** | **INSP O2 %** |
| --- | --- | --- | --- | --- | --- | --- | --- | --- | --- |
| **Full dataset training** | **mean** | 36.64 | 127.89 | 87.36 | 19.65 | 93.66 | 0.02 | 0.89 | 22.68 |
|  | **std** | 0.61 | 22.77 | 16.02 | 3.34 | 3.54 | 0.15 | 1.71 | 7.50 |
|  | **min** | 30.1 | 49 | 26 | 1 | 50 | 0 | 0 | 21 |
|  | **median** | 36.6 | 125 | 87 | 19 | 94 | 0 | 0 | 21 |
|  | **max** | 41.9 | 279 | 255 | 98 | 100 | 3 | 15 | 100 |
| **Full dataset validation** | **mean** | 36.63 | 128.26 | 85.73 | 19.73 | 93.75 | 0.01 | 0.95 | 23.72 |
|  | **std** | 0.53 | 21.80 | 15.65 | 3.42 | 3.17 | 0.11 | 2.02 | 9.22 |
|  | **min** | 32.0 | 43 | 39 | 8 | 37 | 0 | 0 | 21 |
|  | **median** | 36.6 | 126 | 86 | 19 | 94 | 0 | 0 | 21 |
|  | **max** | 40.9 | 252 | 258 | 60 | 100 | 3 | 15 | 100 |
| **Annotated dataset training** | **mean** | 36.65 | 125.63 | 91.15 | 20.41 | 92.98 | 0.03 | 1.49 | 27.39 |
|  | **std** | 0.64 | 23.83 | 17.34 | 4.15 | 3.85 | 0.23 | 2.18 | 13.50 |
|  | **min** | 32.0 | 51 | 26 | 1 | 50 | 0 | 0 | 21 |
|  | **median** | 36.6 | 123 | 92 | 20 | 93 | 0 | 1 | 21 |
|  | **max** | 40.4 | 278 | 230 | 96 | 100 | 3 | 15 | 100 |
| **Annotated dataset validation** | **mean** | 36.64 | 123.69 | 90.53 | 20.43 | 92.82 | 0.02 | 1.41 | 24.04 |
|  | **std** | 0.65 | 21.77 | 16.37 | 3.99 | 3.81 | 0.16 | 2.18 | 9.41 |
|  | **min** | 34.1 | 52 | 39 | 2 | 50 | 0 | 0 | 21 |
|  | **median** | 36.6 | 121 | 90 | 20 | 93 | 0 | 0 | 21 |
|  | **max** | 41 | 250 | 185 | 90 | 100 | 3 | 15 | 100 |

**Table S8: Multivariate logistic regression model coefficients for predicting death or intensive care unit admission occurring within 24 hours**

|  | **RAW** | **NEG** | **POS** | **ROLAVG** | **ROLSTD** | **DIFF** | **SLOPECAT** |
| --- | --- | --- | --- | --- | --- | --- | --- |
| **ACVPU** | 0.096 |  |  | 0.070 | 0.070 | -0.002 |  |
| **HEART_RATE** |  | 0.022 | 0.131 | **0.166** | -0.078 | -0.021 | 0.137 |
| **INSP_O2_CAT** | **0.478** |  |  | 0.082 | -0.057 | -0.031 | **0.186** |
| **O2_SATS** | -0.132 |  |  | 0.003 | 0.083 | 0.031 | **0.156** |
| **RESP_RATE** |  | 0.025 | 0.031 | **0.184** | -0.007 | 0.014 | **0.264** |
| **SYSTOLIC_BP** |  | **0.237** | 0.009 | 0.061 | 0.030 | 0.061 | -0.043 |
| **TEMPERATURE** |  | 0.133 | 0.019 | **-0.160** | 0.012 | 0.037 | 0.051 |

Regression coefficients for each of the 38 variables within the multivariate model are shown. Constant term in the model is -4.962. Rows and columns indicate the clinical observation and feature type respectively. Feature types are as follows: RAW = raw value of clinical observation; NEG = number of units below the lower end of the NEWS-2 zero-scoring range; POS = number of units above the upper end of the NEWS-2 zero-scoring range; ROLAVG and ROLSTD = average and standard deviation of the five (minimum three) most recent observations respectively; DIFF = difference from previous observation; SLOPECAT = categorisation of the five (minimum three) most recent values into normal and stable, normal and unstable, outside normal range and stable, outside normal range and improving, or outside normal range and worsening. The eight features with the highest absolute coefficients are highlighted.

**Table S9: Multivariate logistic regression model coefficients for predicting clinically significant deterioration occurring within 4 hours**

|  | **RAW** | **NEG** | **POS** | **ROLAVG** | **ROLSTD** | **DIFF** | **SLOPECAT** |
| --- | --- | --- | --- | --- | --- | --- | --- |
| **ACVPU** | 0.137 |  |  | -0.080 | 0.090 | 0.022 |  |
| **HEART_RATE** |  | 0.043 | **0.488** | **-0.288** | 0.004 | -0.053 | **0.211** |
| **INSP_O2_CAT** | **0.489** |  |  | **-0.221** | 0.039 | 0.003 | 0.099 |
| **O2_SATS** | -0.135 |  |  | -0.082 | 0.040 | 0.007 | 0.041 |
| **RESP_RATE** |  | 0.011 | 0.130 | 0.028 | 0.000 | 0.014 | **0.425** |
| **SYSTOLIC_BP** |  | **0.368** | 0.020 | **0.196** | 0.070 | 0.097 | 0.028 |
| **TEMPERATURE** |  | 0.085 | 0.011 | 0.041 | -0.018 | 0.053 | 0.152 |

Regression coefficients for each of the 38 variables within the multivariate model are shown. Constant term in the model is -3.654. Rows and columns indicate the clinical observation and feature type respectively. Feature types are as follows: RAW = raw value of clinical observation; NEG = number of units below the lower end of the NEWS-2 zero-scoring range; POS = number of units above the upper end of the NEWS-2 zero-scoring range; ROLAVG and ROLSTD = average and standard deviation of the five (minimum three) most recent observations respectively; DIFF = difference from previous observation; SLOPECAT = categorisation of the five (minimum three) most recent values into normal and stable, normal and unstable, outside normal range and stable, outside normal range and improving, or outside normal range and worsening. The eight features with the highest absolute coefficients are highlighted.
